# COVID-19 GPH: tracking the contribution of genomics and precision health to the COVID-19 pandemic response

**DOI:** 10.1186/s12879-022-07219-3

**Published:** 2022-04-25

**Authors:** Wei Yu, Emily Drzymalla, Marta Gwinn, Muin J. Khoury

**Affiliations:** Office of Genomics and Precision Public Health, Office of Science, Centers for Disease Control and Prevention, Atlanta, GA USA

**Keywords:** COVID-19, Database, SARS-CoV-2, Genomics, Precision Public Health

## Abstract

The scientific response to the COVID-19 pandemic has produced an abundance of publications, including peer-reviewed articles and preprints, across a wide array of disciplines, from microbiology to medicine and social sciences. Genomics and precision health (GPH) technologies have had a particularly prominent role in medical and public health investigations and response; however, these domains are not simply defined and it is difficult to search for relevant information using traditional strategies. To quantify and track the ongoing contributions of GPH to the COVID-19 response, the Office of Genomics and Precision Public Health at the Centers for Disease Control and Prevention created the COVID-19 Genomics and Precision Health database (COVID-19 GPH), an open access knowledge management system and publications database that is continuously updated through machine learning and manual curation. As of February 11, 2022, COVID-GPH contained 31,597 articles, mostly on pathogen and human genomics (72%). The database also includes articles describing applications of machine learning and artificial intelligence to the investigation and control of COVID-19 (28%). COVID-GPH represents about 10% (22983/221241) of the literature on COVID-19 on PubMed. This unique knowledge management database makes it easier to explore, describe, and track how the pandemic response is accelerating the applications of genomics and precision health technologies. COVID-19 GPH can be freely accessed via https://phgkb.cdc.gov/PHGKB/coVInfoStartPage.action.

**Supplementary Information:**

The online version contains supplementary material available at 10.1186/s12879-022-07219-3.

## Background

The COVID-19 pandemic, caused by SARS-CoV-2, broke out at the start of 2020 [1]. The global scientific community responded with extraordinary effort, sharing information in online databases, preprints, and scientific publications. Based on PubMed and preprint server searches, more than 200,000 scientific articles and preprints were published during two years of the pandemic. They report the results of basic, clinical, and population-based investigations, ranging from studies of the virus itself to the global impact of the pandemic on health, economics, and daily life. Rapid growth of the scientific literature on COVID-19 makes it difficult for scientists, clinical and public health professionals, and the community in general to keep up databases such as LitCovid [2] and the World Health Organization COVID-19 database [3], are a key resource for researchers, policy-makers, and the public.

Genomics and data science—including computational methods often referred to as machine learning or artificial intelligence—have been instrumental in many aspects of research on COVID-19. These methods have provided insights into SARS-CoV-2 and how it evolves and spreads in populations, as well as susceptibility to COVID-19 infection, risk of severe outcomes, and role of COVID-19 treatments. Genomic surveillance has documented the emergence and spread of the omicron and delta SARS-CoV-2 variants in Denmark [4] as well as the unique mutations that differ between these two variants [5]. Other studies have described the role of human genetic polymorphisms in COVID-19 susceptibility [6], the upregulation of proinflammatory cytokine genes in severe COVID-19 patients [7], and the toxicity profiles of 90 possible COVID-19 treatments using machine learning [8].

To track and provide easier access to the application of genomics and precision health in the COVID-19 response, the CDC Office of Genomics and Precision Public Health launched the COVID-19 Genomics and Precision Health knowledge management system and database (COVID-19 GPH) on April 1, 2020. COVID-19 GPH is a component of the Public Health Genomics and Precision Health Knowledge Base (PHGKB). PHGKB features a suite of curated and continuously updated, searchable databases of published scientific literature, CDC resources, and other materials that address the translation of genomics and precision health discoveries into improved health care and disease prevention [9]. Two databases that capture the broad spectrum of biomedical research on COVID-19 have been established by separate groups at the US National Institutes of Health (NIH): LitCOVID at the National Library of Medicine [2], and the iSearch COVID-19 Portfolio at the Office of Portfolio Analysis [10]. In contrast to these databases, COVID-19 GPH was developed to select a subset of the technology-intense scientific literature on COVID-19 that is most relevant to public health and population medicine. Because COVID-19 GPH is curated, users can quickly identify information related to genomics and precision public health without having to compose a complex search query. COVID-19 GPH also links to news, reports, and other relevant information from CDC, NIH and other public health organizations, all updated daily. Thus, in addition to a searchable archive of scientific literature, COVID-19 GPH offers an easily accessible, online update that helps users keep abreast of the latest developments. Here we describe this unique database and its contribution to organizing the rapidly expanding knowledge base on COVID-19.

### Construction and content

#### Implementation

COVID-19 GPH is a web-based application based on J2EE technology [11] with Java open-source frameworks including Hibernate [12] and Strut [13]. As a component of the PHGKB system, COVID-19 GPH has been built on and integrated into the overall architecture of PHGKB described previously [14, 15].

#### Data retrieval

**Fig. 1:**
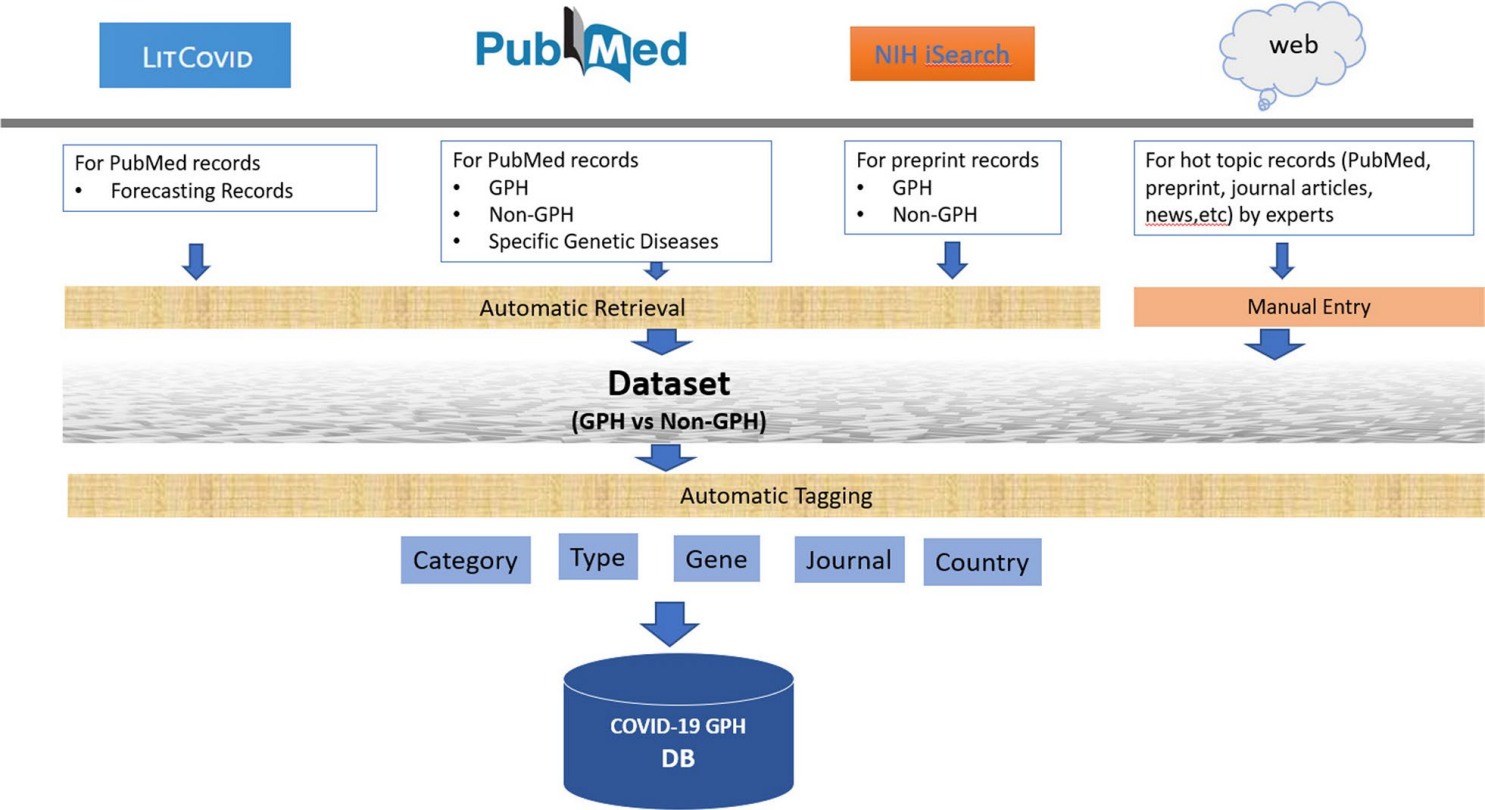
COVID-19 GPH data retrieval and curation processes

Data are collected mainly from PubMed, the NIH iSearch COVID-19 Portfolio [10], LitCovid [2], and common media sources by an automatic retrieval and text mining strategy [15], combined with manual curation by domain experts at the Centers for Disease Control and Prevention (CDC) (Fig. 1). Data are retrieved by four main approaches. First, the scientific publications are retrieved from PubMed daily by an automated script using NCBI Eutils [16] using two specifically designed queries (Additional file 1: Appendix I). Second, we use the same queries to search the NIH iSearch COVID-19 Portfolio website and download records retrieved in spreadsheet format which are subsequently uploaded to the database using an automatic script. Third, we automatically retrieve records classified to the epidemic forecasting category in the LitCovid database using the LitCovid RSS feed. Finally, CDC staff selects online news and other reports from our weekly horizon scan for the Genomics Health Impact Update [17] and Advanced Molecular Detection Clips [18] and other sources. The inclusion and exclusion criteria for these weekly scans are described in detail in the Additional file 1: Appendix II. The curation pipelines include a series of computer scripts for scheduled automatic data retrieval and uploading, along with a web-based curation interface that CDC domain experts use to select and curate important news, reports, and articles. The PubTator web service is used to annotate gene information in PubMed records. A text mining technique [14] is used to identify and standardize the country information associated with the authors in PubMed records. All data selection processes are performed daily. To prevent potential record duplication through multiple retrieval processes, we use a de-duplication mechanism based on unique PubMed IDs or publication titles.

#### Data classification

Data are classified into two main groups: Genomics Precision Health and Non-Genomics Precision Health (Additional file 1: Appendix II). They are then further classified automatically into 10 different categories: eight based on the PubTator [19] classifier in the LitCovid database [2] (mechanism, treatment, prevention, diagnosis, forecasting, surveillance, transmission) by querying and parsing LitCovid RSS feeds, and three created using text mining scripts (vaccine, variant, health equity) using keyword searching (keywords in Additional file 1: Appendix III). Data are also classified to 12 topics with their own sub-databases in PHGKB (Cancer; Diabetes; Heart, Lung, Blood and Sleep Diseases; Rare Diseases; Health Equity; Family Health History; Reproductive and Child Health; Pharmacogenomics; Neurological Disorders; Primary Immune Deficiency; Environmental Health).

#### Evaluation of data retrieval performance

To validate our automated data retrieval process, we generated a 499-item random sample from the LitCovid database on April 23, 2021. These records were screened automatically as shown in Fig. 1 and classified as positive (included in the database) or negative (excluded from the database). The automatic query included 55 articles and excluded 444 articles. At the same time, two domain experts independently reviewed the same 499 records manually and classified them according to the database inclusion and exclusion criteria. They discussed all 23 instances of disagreement and arrived at a final classification by consensus. The experts included 50 articles and excluded 449 articles. The performance of the automated retrieval process was evaluated by calculating its specificity and sensitivity, using expert classification as the gold standard. The automatic curation process has an estimated sensitivity of 0.82 and specificity of 0.97 for PubMed articles (Table 1).

**Table 1:** Performance evaluation of the automatic curation process (ACP)

	Experts		Sum
	Positive	Negative	
ACP			
Positive	41	14	55
Negative	9	435	444
Sum	50	449	499

Specificity = 0.97, Sensitivity = 0.82

#### User interface and features

**Fig. 2:**
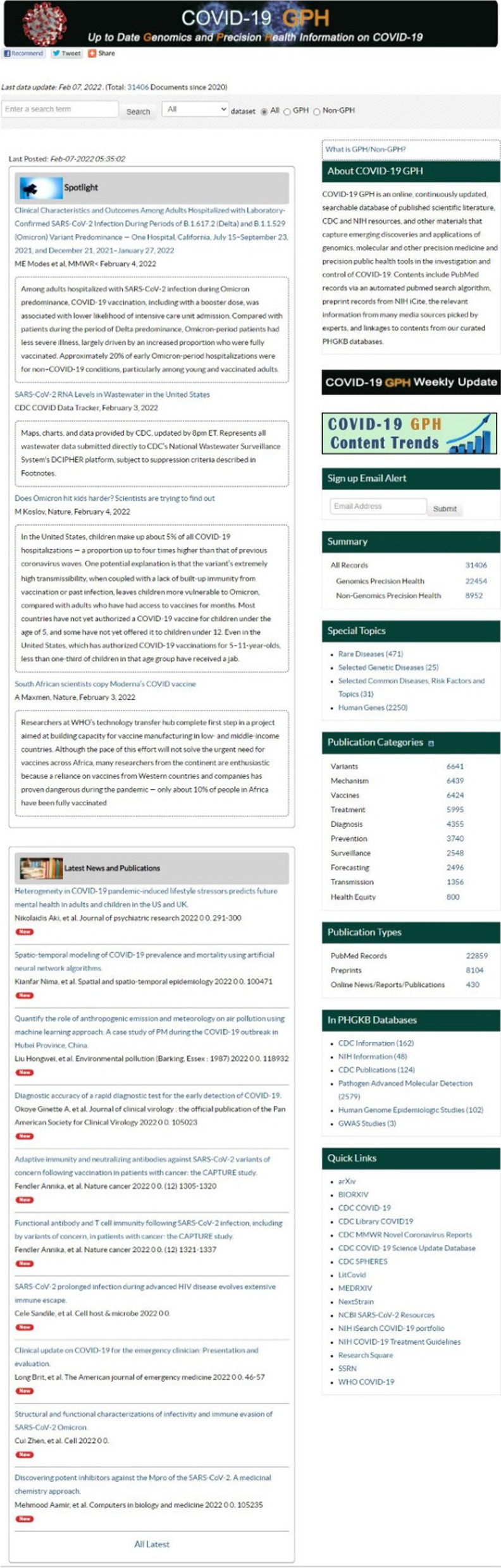
The screenshot of COVID-19 GPH landing page

The COVID-19 GPH web-based user interface is shown in Fig. 2. The landing page of the site provides two main sections that list important publications picked by a CDC domain expert (Spotlight) and the most recent records added to the database (Latest News and Publications). Summary statistics are on the left side of the page. The user interface allows users to perform a free text search on any topic. The search results can be further stratified by five filters (Country, Journal, Gene, Publication Type and Publication Category). The filtering process can be repeated until a desired search result is achieved. Users can also perform a search on sub-datasets for 10 special topics in PHGKB. Two graphs can be drawn dynamically to summarize the search results: (1) Distribution of Publications by Month and (2) Distribution of Publication by Category. Users also can sign up for a COVID-19 GPH Weekly Update email newsletter that includes COVID-19 related items selected by CDC staff in these categories: Pathogen and Human Genomics Studies, Non-Genomics Precision Health Studies and News/Reviews/Commentaries.

### Utility and discussion

#### Description

COVID-19 GPH is an open access, online database containing links to original studies, reviews, commentaries, and news relevant to genomics, machine learning, or the use of big data in COVID-19 research. Although most records are extracted from PubMed, the database also contains preprints as well as selected online news, reports, and publications (Table 2). Included articles reference 845 human genes, with *ACE2* being the most common.

**Table 2:** Number of articles in COVID-19 GPH from each source

Publication type	Number^a^
PubMed	22,983
Online news/reports/publications	431
Preprint	8172

^a^Number as of February 11, 2022

**Fig. 3:**
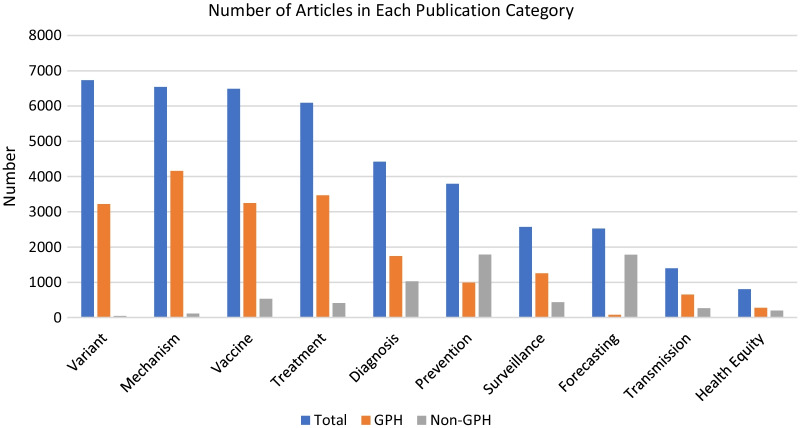
Number of articles in each publication category. Numbers on February 11, 2022. The definitions for the publication categories are: mechanism: underlying cause(s) of COVID-19 infections and transmission and possible drug mechanism of action; transmission: characteristics and modes of covid-19 transmissions, such as human-to-human, diagnosis: disease assessment through symptoms, test results, and radiological features; prevention: prevention, control, response, and management strategies; forecasting: modelling and estimating the trend of COVID-19 spread; health equity: relevant to health equity and search terms are derived from a list provided by the Association for Territorial Health Officials which include terms such as diversity, health disparities, and others; vaccine: relevant to vaccine development, evaluation, implementation and impact; variant: relevant to SARS-CoV-2 variants and their impact on public health; surveillance: relevant to SARS-CoV-2 public health surveillance and tracking

The database contains information on the surveillance, investigation, diagnosis, treatment, prevention, and control of COVID-19. The contents are divided into two main sections, Genomics Precision Health (GPH) and Non-Genomics Precision Health (Non-GPH). GPH contains literature focused on applications of pathogen and human genomics. The literature in Non-GPH relates to the use of big data, data science, digital health, machine learning, predictive analytics and forecasting methods. As of February 11, 2022, the database contains 31,597 articles (22,597 GPH, 9,000 Non-GPH). Articles in both categories may be classified into one or more of 11 publication categories (Table 3). These categories are not mutually exclusive, and an article may be assigned to more than one. In the entire database, the largest category is “Variants” (n = 6735) and the smallest is “Health Equity” (n = 804); however, the relative sizes of these categories differ between the GPH and non-GPH groups (Fig. 3). Some common topics among articles included in the database are listed in Table 4, along with examples [16–29]. We estimated the fraction of scientific literature on selected for COVID-19 GPH by dividing the number of PubMed records in COVID-19 GPH by the number of PubMed records in LitCovid: 22983/221241 (10%) based on the data retrieved on February 11, 2022.

**Table 3:** Publication category definitions

Publication category	Description
Categories annotated by LitCovid from NCBI NIH [1]	
Mechanism	Underlying cause(s) of covid-19 infections and transmission and possible drug mechanism of action
Transmission	Characteristics and modes of covid-19 transmissions, such as human-to-human
Diagnosis	Disease assessment through symptoms, test results, and radiological features
Prevention	Prevention, control, response, and management strategies
Forecasting	Modelling and estimating the trend of covid-19 spread
Categories annotated by the text mining tool from CDC PHGKB	
Health Equity	Relevant to health equity. Search terms are derived from a list provided by the Association for Territorial Health Officials which include terms such as diversity, health disparities, and others
Vaccine	Relevant to vaccine development, evaluation, implementation, and impact
Variant	Relevant to SARS-CoV-2 variants and their impact on public health
Surveillance	Relevant to SARS-CoV-2 public health surveillance and tracking

**Table 4:** Selected topics in the COVID-19 GPH database, with examples

Section	Topic	Article name	Summary
GPH	Variants of SARS-CoV-2	Genomic Analysis and Lineage Identification of SARS-CoV-2 Strains in Migrants Accessing Europe Through the Libyan Route [16]	This study sequenced SARS-CoV-2 strains to determine the genetic variation and lineage of the virus in migrants [16]
		Rapid Emergence and Epidemiologic Characteristics of the SARS-CoV-2 B.1.526 Variant [17]	This MMWR discusses the epidemiology B.1.526 variant, which has a E484K mutation, and compares the severity of disease resulting from B.1.526 to other SARS-CoV-2 strains [17]
	Role of human genetic variation in COVID-19 susceptibility and severity	A cluster of differentiation 14(CD14) polymorphism (C-159T rs2569190) is associated with SARS-CoV-2 infection and mortality in the European population [20]	This study examined the association between the infection rate for COVID-19 in countries and the T allele frequency for rs2569190 in the country’s population [20]
		The influence of IFITM3 polymorphisms on susceptibility to SARS-CoV-2 infection and severity of COVID-19 [21]	This study compared positive and negative SARS-CoV-2 cases in order to determine whether are genotypic differences between the cases for SNPs within *IFITM3* [21]
	Efficacy of SARS-CoV-2 vaccines^a^	Efficacy of NVX-CoV2373 Covid-19 Vaccine against the B.1.351 Variant [22]	Different strains of SARS-CoV-2 have varying genotypes. As a result, the efficacy of a vaccine may vary for different strains. This study provides evidence for the efficacy of the NVX-CoV2373 vaccine against a specific strain, B.1.351 [22]
		Antibody Response to 2-Dose SARS-CoV-2 mRNA Vaccine Series in Solid Organ Transplant Recipients [23]	mRNA vaccines use viral mRNA to generate immunity. This study examines the antibody response in patients who received solid organ transplants after receiving up to 2 doses of a mRNA vaccine for COVID-19 [23]
	Impact of COVID-19 on people with genetic diseases	BRCA testing in a genomic diagnostics referral center during the COVID-19 pandemic [18]	Mutations in *BRCA1* and *BRCA2* are associated with hereditary breast and ovarian cancer syndrome. This article provides evidence for the decrease in BRCA testing during the COVID-19 pandemic [18]
		SARS-CoV-2 infection associated with hepatitis in an infant with X-linked severe combined immunodeficiency [19]	A case report of 11 week old infant with X-linked severe combined immunodeficiency, a genetic disease resulting from mutations in *IL2RG*, who tested positive for COVID-19 [19]
Non-GPH	Epidemic modeling of COVID-19	Estimated transmissibility and impact of SARS-CoV-2 lineage B.1.1.7 in England [24]	This study developed a mathematical model to predict the reproduction number, resulting hospitalizations, and resulting deaths for the B.1.1.7 COVID-19 variant [24]
		Modeling COVID-19 Pandemic with Hierarchical Quarantine and Time Delay [25]	This study predicts the spread of COVID-19 using a SIDQR model and the effect of hierarchical quarantine on the spread of the virus [25]
	Machine learning applied to COVID-19 data	COVID-Classifier: an automated machine learning model to assist in the diagnosis of COVID-19 infection in chest X-ray images [26]	This study to test the accuracy of a machine learning model to distinguish between COVID-19 cases, pneumonia cases, and normal cases using chest X-ray images [26]
		Application of Artificial Intelligence in COVID-19 Pandemic: Bibliometric Analysis [27]	This review determines the research trends and publication patterns for using artificial intelligence for COVID-19 [27]
	Big data analysis	Public Perception of COVID-19 Vaccines through Analysis of Twitter Content and Users [28]	This study analyzed about 2.4 million tweets from about 1 million users in order to determine the general attitude of the public toward COVID-19 vaccination [28]
		IgM anti-SARS-CoV-2-specific determination: useful or confusing? Big Data analysis of a real-life scenario [29]	This study analyzed a laboratory database, about 209,408 samples and tests, to determine the clinical utility of IgM detection in slowing the spread of COVID-19 [29]

^a^mRNA vaccines or vaccines concerning SARS-CoV-2 variants

**Fig. 4:**
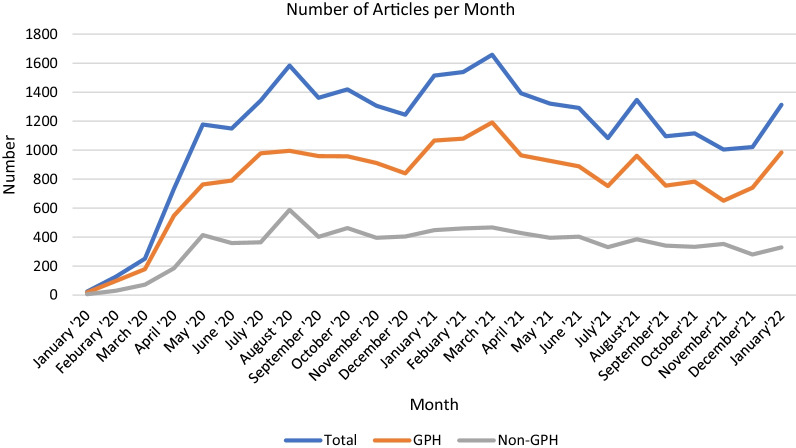
Number of articles per month for all articles, GPH articles only, and non-GPH articles only

**Fig. 5:**
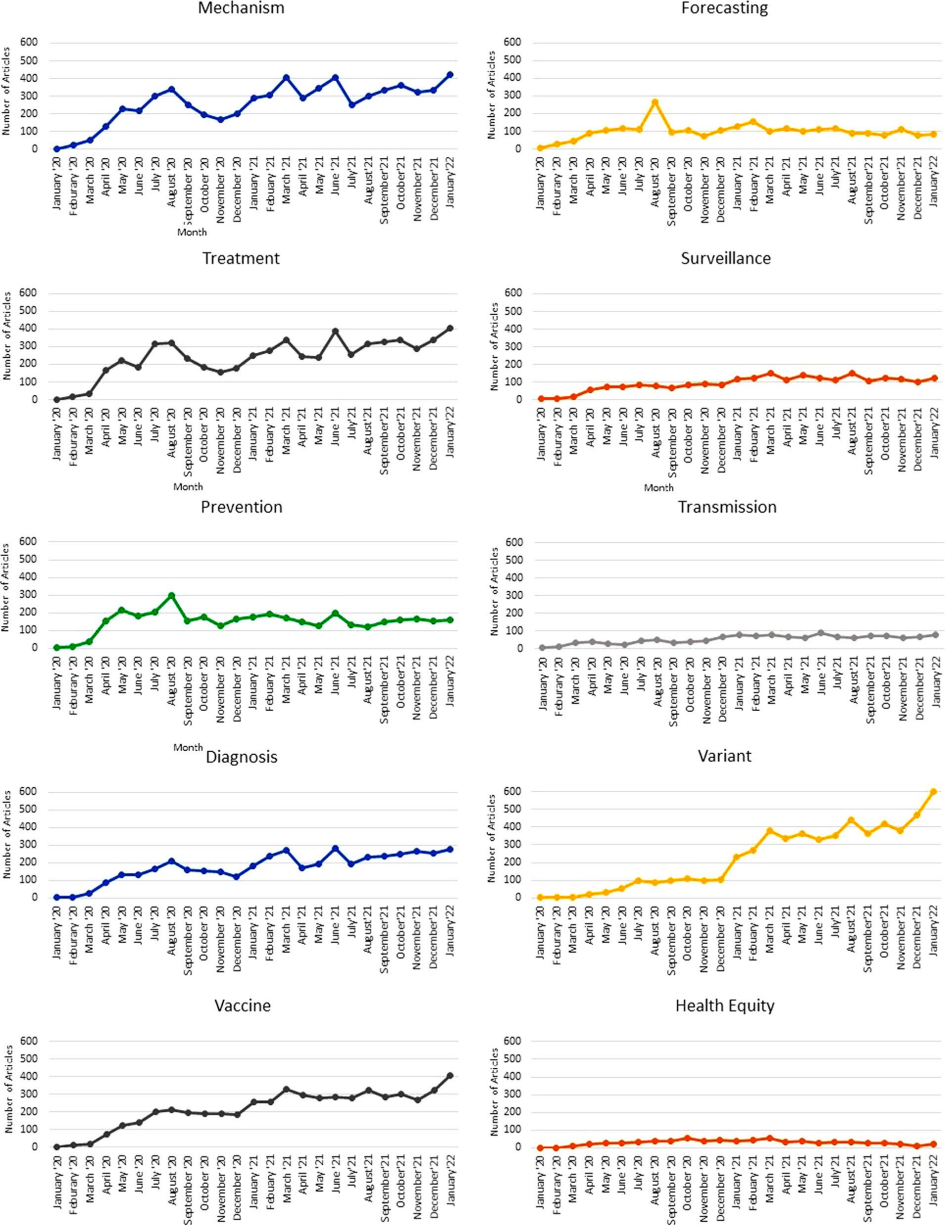
Number of articles per month by publication category

The database can be used to analyze publication trends by month (Fig. 4). After increasing rapidly in early 2020, the number of articles published per month has generally remained between 1000 and 1700. (Note that because of processing time at PubMed, the number for January 2022 may be incomplete). Trends by category tend to be consistent overall, except for prevention and forecasting which peaked 2020 (Fig. 5). Articles in several other categories (variants, vaccine, mechanism, and diagnosis) generally increased in 2021 (Fig. 5).

**Fig. 6:**
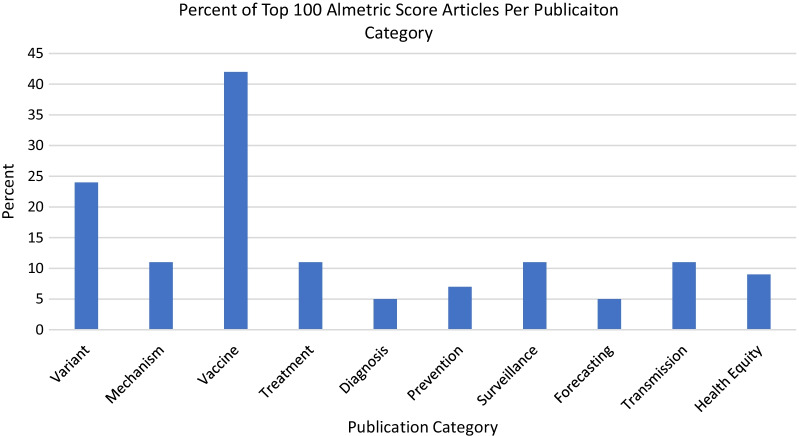
Percent of articles per publication category with 100 highest Altmetric scores

For each PubMed publication, the database also captures the Altmetric score, a numerical value indicating the amount of attention an article has received [30]. Of the articles with the top 100 Altemetric scores, the vaccine category accounted for the largest share (26%) and the variant category was second (12%) (Fig. 6).

The database simplifies the search for COVID-19 and certain rare diseases, including articles related to 471 of the approximately 7,000 rare diseases on the NIH Genetic and Rare Diseases Information Center website [31]. Users can also search for articles common to COVID-19 GPH and other specialized PHGKB databases. Of the specialized databases, rare disease has the most overlap, 6811 articles, with COVID-19 GPH while Family Health History shares the least number of articles, 10 (Table 5).

**Table 5:** Comparison of major open-access COVID-19 scientific publication databases

Category	All	GPH	Non-GPH
Rare Disease	6811	5994	817
Heart, Lung, Blood, and Sleep Disorders	3689	2443	1246
Environmental Health	1839	1195	644
Pharmacogenomics	1295	1165	130
Health Equity	802	448	354
Cancer	690	586	104
Neurological Disorders	623	483	140
Diabetes	514	359	155
Reproductive and Child Health	448	382	66
Primary Immune Deficiency	362	358	4
Family Health History	10	8	2

## Discussion

The COVID-19 pandemic has produced a surge of original studies, reviews, commentaries, and news available to the scientific community and the public. Two broad emerging technologies including genomics (pathogen and human) and precision health (big data, machine learning, artificial intelligence, and predictive analytics) have been widely used in COVID-19 research, surveillance and response. However, the contribution and evolution of these technologies may be difficult to discern in the midst of the rapid growth of COVID-19 publications. COVID-19 GPH is a an online, continuously updated database that captures the evolving contribution of genomics and digital technologies to the COVID-19 response. Its domain encompasses a wide range of topics, from phylogenetic analysis of SARS-CoV-2 to artificial intelligence for COVID-19 diagnosis. Overall, the contents of COVID-19 GPH represent about 10% of all the COVID-19 literature available from PubMed. Databases such as LitCovid, the World Health Organization COVID-19 database, iSearch COVID-19 Portifolio, CORD-19 and PubMed are comprehensive sources for published scientific articles on COVID-19.

The COVID-19 GPH database is designed for researchers interested specifically in the domains of genomics and precision health, providing several key advantages over more general COVID-19 databases or PubMed. The data are updated daily from multiple sources. The web interface allows users to search the data in a free-text manner and to stratify the search results with meaningful, pre-classified categories and types. For example, users can search by country, journal, gene, publication category, or publication type (PubMed, Preprint, or other), within either the GPH or Non-GPH category or overall. The database also allows users to follow publication trends and monitor online impact.

The emerging fields of precision medicine and precision public health are driven by advances in genomics and digital technologies [32]. These approaches have found novel and urgent applications in the response to the COVID-19 pandemic and catalyzed international scientific collaboration. For example, international collaboration on pathogen genomics has been crucial for monitoring the emergence of SARS-CoV-2 variants [33]. The COVID-19 Host Genetics Initiative has organized researchers from many countries to study human genetic variation in relation to COVID-19 [34]. Beyond genomics, machine learning has played a role in the COVID-19 response by forecasting disease spread, monitoring public health recommendation adherence, diagnosis, and health equity [35, 36]. For example, using machine learning, a study in the United States was able to identify a disparity for COVID-19 infection and mortality for minority populations [37].

To our knowledge, ours is the only database focused on genomic and precision health for COVID-19. Although the combination of computer and manual curation processes improves quality, it is not perfect; our validation study, which found sensitivity of 0.82 and specificity of 0.97, was limited to PubMed records. We plan to rerun the validation study at the end of the year, after accumulating more data. Our classification by categories is also limited; eight of eleven categories are assigned by LitCovid and thus apply only to PubMed records. Other records are eligible only for the three categories we assign by keyword (vaccine, variant, and health equity), which increases the relative proportions of articles in these categories. In the future, we intend to conduct additional studies to explore the acceptability and functionality of the database for researchers interested in genomics and precision public health in relation to COVID-19.

## Conclusions

COVID-19 GPH is a continuously updated, online database that captures publications describing the applications of genomics and digital technologies to control of the COVID-19 pandemic. Compared with larger, more wide-ranging databases, it simplifies searching and offers users additional tools for filtering and displaying search results, including charts to display trends over time.

## Supplementary Information

**Additional file 1: Appendix I.** PubMed complex queries. **Appendix II.** The inclusion and exclusion criteria. **Appendix III.** Keywords for searching categories.

## Data Availability

Data for articles contained in COVID-19 GPH can be found at https://phgkb.cdc.gov/PHGKB/coVInfoStartPage.action.

## Abbreviations

PHGKB: Public Health Genomics and Precision Health Knowledge Base
CDC: Centers for Disease Control and Prevention
NIH: National Institutes of Health
