# Supplementary material for: COVID-19 GPH: tracking the contribution of genomics and precision health to the COVID-19 pandemic response
Source: BMC Infect Dis. 2022 Apr 25;22:402. doi: 10.1186/s12879-022-07219-3 (PMC9035978; doi:10.1186/s12879-022-07219-3)
Supplement: Supplementary file 1 — Additional file 1: Appendix I. PubMed complex queries. Appendix II. The inclusion and exclusion criteria. Appendix III. Keywords for searching categories. [file 12879_2022_7219_MOESM1_ESM.docx]

**Appendix I. PubMed complex queries**

**Genomics Precision Health:**

(((((("SARS-CoV-2"[TIAB]) OR "COVID-19"[TIAB]) OR "2019-nCoV"[TIAB]) OR "Severe Acute Respiratory Syndrome"[TIAB])) AND (((((((((((((((((((((("bioinformatical"[TIAB] OR "bioinformatically"[TIAB]) OR "computational biology"[MeSH Terms]) OR ("computational"[TIAB] AND "biology"[TIAB])) OR "computational biology"[TIAB]) OR "bioinformatic"[TIAB]) OR "bioinformatics"[TIAB])) OR (((((((((((("genetic therapy"[MeSH Terms] OR ("genetic"[TIAB] AND "therapy"[TIAB])) OR "genetic therapy"[TIAB]) OR "genetic"[TIAB]) OR "genetical"[TIAB]) OR "genetically"[TIAB]) OR "genetics"[MeSH Subheading]) OR "genetics"[TIAB]) OR "genetics"[MeSH Terms]) OR "genet"[TIAB]) OR "genets"[TIAB])) OR ((((((("genome"[MeSH Terms] OR "genome"[TIAB]) OR "genomes"[TIAB]) OR "genome s"[TIAB]) OR "genomically"[TIAB]) OR "genomics"[MeSH Terms]) OR "genomics"[TIAB]) OR "genomic"[TIAB])) OR (("genes"[MeSH Terms] OR "genes"[TIAB]) OR "gene"[TIAB])) OR (((((((((("mutate"[TIAB] OR "mutated"[TIAB]) OR "mutates"[TIAB]) OR "mutating"[TIAB]) OR "mutation"[MeSH Terms]) OR "mutation"[TIAB]) OR "mutations"[TIAB]) OR "mutation s"[TIAB]) OR "mutational"[TIAB]) OR "mutator"[TIAB]) OR "mutators"[TIAB])) OR ((((((((((("genotype"[MeSH Terms] OR "genotype"[TIAB]) OR "genotypes"[TIAB]) OR "genotypic"[TIAB]) OR "genotype s"[TIAB]) OR "genotyped"[TIAB]) OR "genotyper"[TIAB]) OR "genotypical"[TIAB]) OR "genotypically"[TIAB]) OR "genotyping"[TIAB]) OR "genotypings"[TIAB]) OR "genotypization"[TIAB])) OR ((((((("genome"[MeSH Terms] OR "genome"[TIAB]) OR "genomes"[TIAB]) OR "genome s"[TIAB]) OR "genomically"[TIAB]) OR "genomics"[MeSH Terms]) OR "genomics"[TIAB]) OR "genomic"[TIAB])) OR ((((((((((("genotype"[MeSH Terms] OR "genotype"[TIAB]) OR "genotypes"[TIAB]) OR "genotypic"[TIAB]) OR "genotype s"[TIAB]) OR "genotyped"[TIAB]) OR "genotyper"[TIAB]) OR "genotypical"[TIAB]) OR "genotypically"[TIAB]) OR "genotyping"[TIAB]) OR "genotypings"[TIAB]) OR "genotypization"[TIAB])) OR ((((((("polymorphic"[TIAB] OR "polymorphics"[TIAB]) OR "polymorphism s"[TIAB]) OR "polymorphism, genetic"[MeSH Terms]) OR ("polymorphism"[TIAB] AND "genetic"[TIAB])) OR "genetic polymorphism"[TIAB]) OR "polymorphism"[TIAB]) OR "polymorphisms"[TIAB])) OR (((((((((((("allel"[TIAB] OR "allele s"[TIAB]) OR "alleleic"[TIAB]) OR "alleles"[MeSH Terms]) OR "alleles"[TIAB]) OR "allele"[TIAB]) OR "allelic"[TIAB]) OR "allelically"[TIAB]) OR "allelism"[TIAB]) OR "allelisms"[TIAB]) OR "allelle"[TIAB]) OR "allelles"[TIAB]) OR "allels"[TIAB])) OR (("variant"[TIAB] OR "variant s"[TIAB]) OR "variants"[TIAB])) OR (((((((((((("allel"[TIAB] OR "allele s"[TIAB]) OR "alleleic"[TIAB]) OR "alleles"[MeSH Terms]) OR "alleles"[TIAB]) OR "allele"[TIAB]) OR "allelic"[TIAB]) OR "allelically"[TIAB]) OR "allelism"[TIAB]) OR "allelisms"[TIAB]) OR "allelle"[TIAB]) OR "allelles"[TIAB]) OR "allels"[TIAB])) OR ((("heterozygote"[MeSH Terms] OR "heterozygote"[TIAB]) OR "heterozygotes"[TIAB]) OR "heterozygotic"[TIAB])) OR ((((("haplotyped"[TIAB] OR "haplotypes"[MeSH Terms]) OR "haplotypes"[TIAB]) OR "haplotype"[TIAB]) OR "haplotypic"[TIAB]) OR "haplotyping"[TIAB])) OR ((("genome-wide association study"[MeSH Terms] OR (("genome wide"[TIAB] AND "association"[TIAB]) AND "study"[TIAB])) OR "genome wide association study"[TIAB]) OR "gwas"[TIAB])) OR "genome wide association"[TIAB]) OR "hereditary"[TIAB])) "

**Non-Genomics Precision Health:**

((((("SARS-CoV"[Title/Abstract]) OR "SARS-CoV-2"[Title/Abstract]) OR "COVID-19"[Title/Abstract]) OR "2019-nCoV"[Title/Abstract]) OR "Severe Acute Respiratory Syndrome"[Title/Abstract]) AND (((((((((((((((((((((("Big data"[Title/Abstract] OR "Data science"[Title/Abstract]) OR "Machine Learning"[Title/Abstract]) OR "Monte Carlo"[Title/Abstract]) OR "Digital Health"[Title/Abstract]) OR "spatial modelling"[Title/Abstract]) OR "Natural Language Processing"[Title/Abstract]) OR "NLP"[Title/Abstract]) OR "naive Bayes"[Title/Abstract]) OR "Data Mining"[Title/Abstract]) OR "high-dimensional"[Title/Abstract]) OR "Artificial Intelligence"[Title/Abstract]) OR "Predictive Analytics"[Title/Abstract]) OR "Digital Health"[Title/Abstract]) OR "Random forest"[All Fields]) OR "Image processing"[All Fields]) OR "Deep Learning"[Title/Abstract]) OR "Neural Network"[Title/Abstract]) OR "Image processing"[Title/Abstract]) OR "Classification model"[Title/Abstract]) OR "Supervised learning"[Title/Abstract]) OR "Support Vector Machine"[Title/Abstract]) OR "SVM"[Title/Abstract])

**Appendix II: The inclusion and exclusion criteria**

**Genomics Precision Health:**

Goal: COVID-19 GPH displays original studies, reviews, commentaries and news relevant to the applications of pathogen and human genomics and advanced molecular detection methods in in the investigation, diagnosis, treatment, prevention, surveillance and control of COVID-19.

Inclusion criteria

- Biological, clinical, or population studies for the host genome, epigenome, genomic-based transcriptomics, and genomic-based proteomics and COVID-19 pertaining to severity, susceptibility, and transmission
- Biological, clinical, or epidemiologic studies for alterations of the host epigenome, genomic-based transcriptomics, or genomic-based proteomics as a result of COVID-19
- Genomic characteristics of COVID-19, including variants, and how severity, susceptibility, testing, and transmission may be affected
- COVID-19 variants, genomic sequencing for novel variants, variant tracking through modeling the mutation rate and spread
- mRNA vaccines for COVID-19; Vaccine efficacy studies, clinical, biological, or epidemiologic, concerning varying strains of COVID-19 or host genotypes
- Molecular detection strategies for COVID-19 including detecting variants using genomics such as serotype
- Treatments for COVID-19 pertaining to genomics such as pharmacogenomics or success of treatments with different variants
- Genetic diseases and COVID-19 severity, susceptibility, and transmission; the effect of COVID-19 on treatment for genetic diseases

Exclusion criteria

- Studies focusing on previous coronaviruses such as SARS or MERS
- Non-genomic based physiological, chemical, or biochemical studies
- Non-genomic based biomarker studies
- Non-mRNA vaccines for COVID-19 not pertaining to genomics

**Non-Genomics Precision Health:**

Goal: COVID-19 non-GPH displays original studies, reviews, commentaries and news relevant to the use of big data, data science, digital health, machine learning, predictive analytics and forecasting methods in the investigation, diagnosis, treatment, prevention, surveillance and control of COVID-19.

Inclusion criteria

- Use of big data methods, artificial intelligence, or machine learning determining COVID-19 characteristics such as severity, susceptibility, and transmission for populations
- The use of artificial intelligence or big data methods for determining proper prevention measures, screening, and spread of COVID-19
- Modeling the outcomes of COVID-19 such as infection, recovery, and fatality rates as well as vaccine impact
- Use of artificial intelligence, machine learning, or big data in the diagnosis/screening of COVID-19
- Drug treatment outcomes for COVID-19 patients accessed through big data methods or predicted by artificial intelligence or machine learning.
- Articles concerning databases for COVID-19 literature

Exclusion criteria

- Studies that focus on GPH (see above)
- Studies focusing on previous coronaviruses such as SARS or MERS

**Appendix III: Keywords for searching categories**

| **Category** | **Searching Keywords** |
| --- | --- |
| **Vaccine** | **Vaccine**  **Vaccination**  **Immunization**  **Vaccines** |
| **Variant** | Variant  Variants |
| **Health Equity** | **health equity**  **social determinants of health**  **adverse childhood experiences**  **minity groups**  **ethnic groups**  **cultural competency**  **culturally competent care**  **cultural diversity**  **transcultural nursing**  **vulnerable populations**  **socioeconomic facts**  **health status disparities**  **healthcare disparities**  **social justice**  **social class**  **health impact assessment**  **medically uninsured**  **adverse childhood experience***  **national class standards**  **community based participaty research**  **culturally competent**  **culturally AND appropriate**  **linguistically AND inappropriate**  **cultural competency**  **environmental justice**  **health disparit***  **health in all policies**  **health inequit***  **health inequalit***  **high risk population***  **social determinants of health**  **social gradient of health**  **socioeconomic status**  **structural inequality**  **underinsured**  **under-resourced**  **disadvantaged**  **vulnerable populations** |
| **Surveillance** | **surveillance**  **tracking** |
